# Insights into the Structural Basis of Antibody Affinity Maturation from Next-Generation Sequencing

**DOI:** 10.3389/fimmu.2018.00117

**Published:** 2018-02-01

**Authors:** Arjun K. Mishra, Roy A. Mariuzza

**Affiliations:** ^1^W. M. Keck Laboratory for Structural Biology, Institute for Bioscience and Biotechnology Research, University of Maryland, Rockville, Rockville, MD, United States; ^2^Department of Cell Biology and Molecular Genetics, University of Maryland, College Park, College Park, MD, United States

**Keywords:** antibody, affinity maturation, somatic hypermutation, HIV-1, structural biology, next-generation sequencing

## Abstract

Affinity maturation is the process whereby the immune system generates antibodies of higher affinities during a response to antigen. It is unique in being the only evolutionary mechanism known to operate on a molecule in an organism’s own body. Deciphering the structural mechanisms through which somatic mutations in antibody genes increase affinity is critical to understanding the evolution of immune repertoires. Next-generation sequencing (NGS) has allowed the reconstruction of antibody clonal lineages in response to viral pathogens, such as HIV-1, which was not possible in earlier studies of affinity maturation. Crystal structures of antibodies from these lineages bound to their target antigens have revealed, at the atomic level, how antibodies evolve to penetrate the glycan shield of envelope glycoproteins, and how viruses in turn evolve to escape neutralization. Collectively, structural studies of affinity maturation have shown that increased antibody affinity can arise from any one or any combination of multiple diverse mechanisms, including improved shape complementarity at the interface with antigen, increased buried surface area upon complex formation, additional interfacial polar or hydrophobic interactions, and preorganization or rigidification of the antigen-binding site.

## Introduction

The ability of the humoral immune system to generate high-affinity binders for virtually any antigen is predicated on its capacity to produce large repertoires of antibodies encompassing a vast array of specificities and to then select members of this repertoire with high affinity for a particular immunogen (1–3). The extensive sequence diversity of antibody molecules derives from several sources: (i) combinatorial diversification, whereby two sets of light (L) chain gene segments, V_L_ and J_L_, and three sets of heavy (H) chain gene segments, V_H_, D, and J_H_, rearrange to produce functional variable (V) regions; (ii) imprecise joining of these gene segments; and (iii) somatic hypermutation, by which point mutations, as well as insertions and deletions (indels), are introduced throughout the sequences encoding L and H chains (4). B cells expressing antibodies with improved affinity are better equipped to compete for antigen and thus receive signals that result in preferential expansion and further antibody sequence diversification *via* additional rounds of somatic hypermutation (1–3). Through this rapid evolutionary process of mutation and selection, antibody affinity typically improves 10- to 5,000-fold during the course of an immune response, bolstering host defense.

Recent advances in next-generation sequencing (NGS) have revolutionized the analysis of antibody repertoires by dramatically increasing sample depth compared to previous low-throughput methods (5). In addition, new methods have been developed for single-cell sequencing, which allow large-scale determination of paired L and H chains. These advances, in conjunction with computational tools for reconstituting antibody clonal lineages, can provide a genetic record for the evolutionary processes of recombination and somatic hypermutation in immune responses to specific microbial pathogens. Crystal structures of affinity-matured and germ line antibodies from such lineages in complex with their target antigens have produced new insights into the molecular basis of affinity maturation.

Here, we first review our understanding of the basic biophysical principles underlying affinity maturation before the arrival of NGS. We then summarize what studies of bulk B cell populations by NGS have taught us about the general features of antibody repertoire selection. Finally, we discuss structural studies of reconstructed antibody clonal lineages with special emphasis on the immune response to HIV-1, which has so far benefited most from the application of NGS and single-cell analysis to better understanding affinity maturation (6) (Table 1).

**Table 1:** Structural studies of antibody clonal lineages reconstructed using next-generation sequencing.

Antibody lineage	Specificity	PDB code	Reference
CH58	HIV-1 envelope glycoprotein (Env) gp120	4HPO	(7)
		4RIS, 4RIR	(8)

VRC01	HIV-1 Env gp120	4JPV, 4JPW, 4LSP, 4LSQ, 4LSR, 4LSS, 4LST, 4LSU, 4LSV	(9)
		5F7E, 5FA2, 5FEC, 5IGX, 5I90	(10)
		4JPK, 4JPI	(11)

VRC03	HIV-1 Env gp120	5JOF, 5JXA	(12)

CH103	HIV-1 Env gp120	4JAM, 4JAN	(13)
		4QHK, 4QHL, 4QHM, 4QHN	(14)

PGT121	HIV-1 Env gp120	4NCO	(15)
		4R26, 4R2G	(16)
		5CEX, 5CEY, 5CEZ	(17)

PCT64	HIV-1 Env gp120	5FEH	(18)

CAP256	HIV-1 Env gp120	4OCR, 4OCS, 4OCW, 4OD1, 4OD3, 4ODH, 4ORG	(19)

ANC195	HIV-1 Env gp120	5CJX	(20)

C65	Influenza A virus HA	4HK0, 4HK3, 4HKB, 4HKX	(21)

HV6-1 + HD3-3	Influenza A virus HA	5K9J, 5K9K, 5K9O, 5K9Q, 5KAN, 5KAQ	(22)

## Studies of Affinity Maturation Prior to NGS

Before the advent of NGS, a number of structural studies were carried out comparing affinity-matured antibodies and their putative germ line precursors bound to the same antigen. In studies involving small molecules (haptens) such as phenyloxazolone and nitrophenyl phosphonate, rather than proteins, it was found that somatic mutations in complementarity-determining region (CDR) residues directly or indirectly implicated in binding hapten permit the formation of additional hydrogen bonds, electrostatic interactions, and van der Waals contacts (23–26). A particularly revealing case involved the matured 48G7 antibody, which binds nitrophenyl phosphonate ~3,000-fold more tightly than its germ line counterpart 48G7g (26). Large changes in the conformation of the antigen-binding site (paratope) of 48G7g were observed upon hapten engagement by this germ line antibody, whereas the free and hapten-bound forms of affinity-matured 48G7 showed few structural differences. Thus, affinity maturation in this case appeared to be driven largely by a mechanism of preorganizing the paratope into a conformation favorable for binding its hapten ligand (26). Such conformational preorganization was accompanied by a decrease in the flexibility of the paratope during the maturation process, which may increase specificity for the target antigen while reducing the possibility of cross-reactivity with other antigens, including self-antigens (27–29). The antibody maturation process appears to simultaneously select for both higher binding affinity and increased thermodynamic stability. In a study of matured antibody 93F3, which recognizes a small hapten, somatic mutations in the paratope that increased affinity were found to reduce the melting temperature of 93F3 compared to its germ line precursor. However, the destabilizing effects of these mutations were compensated by additional somatic mutations in the V_L_/V_H_ interface, distal to the paratope (29).

The first structural study of the maturation of an antibody response to a protein antigen, instead of a hapten, involved a set of closely related antibodies specific for hen egg white lysozyme (HEL) (30). These antibodies represented different stages of affinity maturation, whereby the number of somatic mutations correlated with increasing affinity. Surprisingly, improved affinity could not be attributed to the formation of additional hydrogen bonds or salt bridges or to an increase in total buried surface area. Instead, affinity maturation resulted mainly from burial of increasing amounts of hydrophobic surface at the expense of polar surface, accompanied by improved shape complementarity at the V_H_–HEL interface. The increase in hydrophobic interactions resulted from highly correlated structural rearrangements in antibody residues at the periphery of the interface with antigen, adjacent to the central energetic hot spot (30). Indeed, the periphery may offer more suitable sites for optimization because these regions are typically more flexible and tolerant to mutations than central sites (12), in agreement with the finding that somatic hypermutation spreads structural diversity generated by V(D)J recombination from central to peripheral regions of the antibody binding site (31).

Collectively, these structural studies showed that increased antibody affinity for small haptens or model protein antigens such as HEL can arise from any one or any combination of several variables, including additional interfacial hydrogen bonds or van der Waals contacts, conformational preorganization of the paratope, improved shape complementarity at the interface with antigen, or increased burial of total or hydrophobic surface area. These same basic strategies, as well as others, govern affinity maturation of antibody responses to biological antigens such as the envelope glycoproteins of HIV-1 and other viral pathogens, as discussed below. We first summarize what NGS of bulk B cell populations has taught us about antibody repertoire selection. We then discuss recent insights into affinity maturation gained from structural studies of antibody clonal lineages that were reconstructed using NGS (Table 1).

## NGS Analysis of Antibody Repertoires in Bulk B Cell Populations

Next-generation sequencing of paired antibody L and H chains combined with computational modeling of antibody structures has been used to profile human antibody repertoire selection and maturation at the population level (5). In the most exhaustive study to date, a comparison of ~55,000 V_L_/V_H_ pairs from naïve B cells with ~120,000 V_L_/V_H_ pairs from antigen-experienced B cells, all isolated from human peripheral blood, showed that V_L_ and V_H_ genes pair in a purely combinatorial fashion with no detectable biases, but that certain V_L_/V_H_ gene pairs are significantly depleted or enriched in the antigen-experienced repertoire compared to the naïve repertoire (32). Repertoire-wide computational structure prediction was carried out to characterize the physiochemical properties of the antibody paratopes. Whereas no appreciable differences in paratope hydrophobicity or solvent-accessible surface area were evident in antigen-experienced versus naïve antibodies, antigen-experienced V_L_CDR3 and V_H_CDR3 amino acid sequences displayed slightly increased positive charge compared to naïve sequences (32). Overall, however, the evolutionary processes of somatic hypermutation and affinity selection that occur in periphery blood did not leave a distinctive physiochemical imprint on the antigen-experienced antibody repertoire, even at the level of CDR3s, which are a major focus for somatic hypermutation. By contrast, bone marrow B cells expressing antibodies with positively charged CDR3 loops undergo preferential elimination at discrete developmental checkpoints before entering the periphery, possibly as a mechanism for reducing the risk of self-reactivity (33).

In a study to determine whether NGS could be used to detect antigen-specific sequences in bulk B cell populations, an analysis of identical CDR3 sequences that were shared by individuals previously vaccinated against *Haemophilus influenzae* type b identified a number of sequences known to be specific for this bacterium (34). Conserved CDR3 sequences were also observed in patients recovering from acute dengue infection (35), indicating convergent antibody evolution in different individuals exposed to the same antigens. In another study, NGS and single-cell sorting of peripheral blood plasmablasts were used to profile the acute antibody response to influenza A vaccination (36). Antibodies able to neutralize the virus were selected bioinformatically from clonal families. These vaccine-induced antibodies contained on average >30 somatic mutations overall. Notably, some antibodies exhibited higher affinities for hemagglutinins (HAs) from prior years’ influenza strains than for the HA of the immunizing strain, suggesting recall of memory B cells expressing antibodies that had previously undergone affinity maturation (36).

## Studies of Affinity Maturation after NGS

Next-generation sequencing coupled with bioinformatics analysis has allowed, for the first time, the reconstruction of antibody clonal lineages and inference of germ line progenitor sequences, neither of which was possible in earlier studies of affinity maturation (37). However, an important caveat is that germ line sequences are predicted sequences that may differ from the true unmutated ancestor sequences. Whereas mutations in V_L_ and V_H_ gene segments can be identified with high confidence, the original V_L_J_L_ and (especially) V_H_DJ_H_ junctional sequences of germ line antibodies are uncertain. In particular, it is impossible to know if insertions or deletions in these sequences took place during V(D)J recombination or were introduced during B cell affinity maturation. As a consequence, statistical methods must be used to infer the most likely unmutated common ancestor for an aligned set of sequences that are taken to be clonally related (37).

As measured by surface plasmon resonance (SPR), a putative germ line precursor of the anti-HIV-1 antibody 2F5, which recognizes the gp41 subunit of the HIV-1 envelope glycoprotein (Env), bound recombinant gp41 with ~500-fold lower affinity than the matured antibody (*K*_D_ = 0.7 µM versus 1.2 nM) (38). Micromolar *K*_D_s were also reported for germ line precursors of broadly neutralizing antibodies (bNAbs) CH01 and CH04, which recognize the V2/V3 quaternary epitope of the gp120 subunit of HIV-1 Env (39). Although seemingly low, such affinities are nevertheless sufficient to trigger affinity maturation of unmutated B cells *in vivo* (40, 41).

By contrast, the putative germ line ancestors of several bNAbs specific for the CD4 binding site of HIV-1 Env (b12, NIH45-46, and 3BNC60) failed to show detectable binding to recombinant Envs, raising the question of how B cell maturation leading to the eventual production of these bNAbs was initiated (42, 43). Moreover, this failure was observed even though the amino acid sequences of the V(D)J junctions of the affinity-matured antibodies were left unchanged in the reconstructed germ line versions. One possibility is that maturation of anti-CD4 binding site bNAb precursors was triggered by non-HIV antigens and that the resultant antibody intermediates serendipitously cross-reacted with Env. More likely, however, interactions between proteins in solution (3D affinity), as measured by SPR or related techniques, differ from those at contacts between two cells or between a cell and a virus (2D affinity) (44). For example, whereas SPR was unable to detect any binding between CD4 and MHC class II (45), the affinity of CD4 for MHC class II on B cells could be measured in 2D using CD4-functionalized supported lipid bilayers (46). Therefore, under physiological conditions, germ line precursors of anti-CD4 binding site bNAbs, expressed on the surface of B cells, might be engaged by membrane-anchored Env on the virion surface or on the surface of infected cells with sufficient affinity to trigger B cell maturation. In support of this idea, the germ line precursors of several anti-influenza HA bNAbs were found to bind to HA only when presented on membranes in the form of cell surface IgMs; as soluble IgGs, these precursors had no detectable affinity for HA (47). In the following sections, we have selected representative examples of affinity maturation in order to illustrate the multiple structural strategies that the antibodies employ to increase potency and breadth of pathogen neutralization.

### Preorganization, Rigidification, and Reorientation

**Figure 1:**
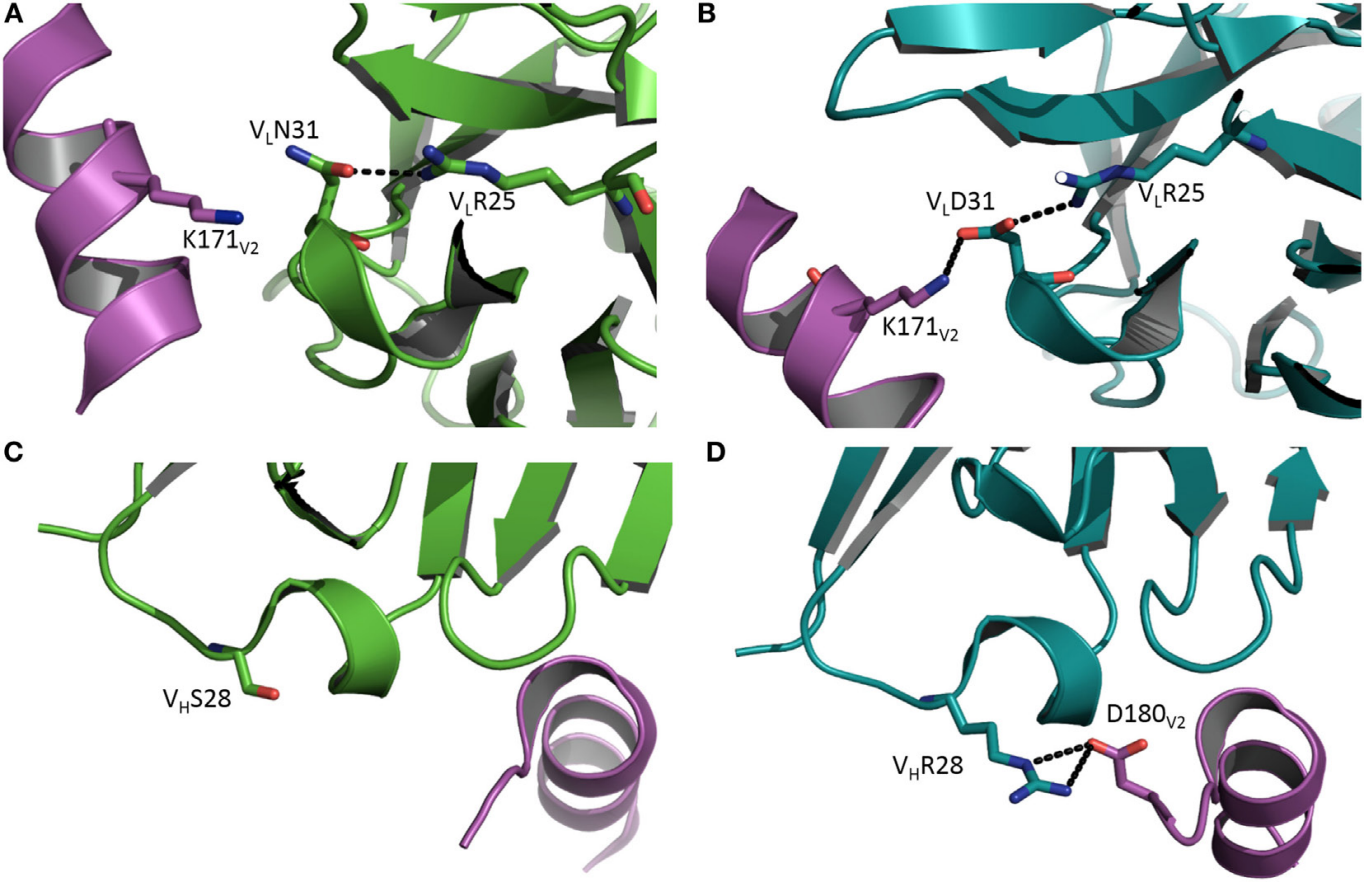
Affinity maturation through formation of additional interactions with antigen. **(A)** Close-up view of the interface between the germ line precursor of antibody CH58 and the V2 peptide of HIV-1 envelope glycoprotein in the vicinity of V2 Lys171 (Protein Data Bank accession code 4RIS) (8). V_L_ is green; V2 is magenta. **(B)** In affinity-matured CH58, the somatic mutation V_L_Asn31→Asp allows formation of a stabilizing salt bridge (dotted black line) to V2 Lys171. V_L_ is teal. **(C)** Close-up of the interface between the germ line precursor of CH58 and the V2 peptide in the vicinity of V_H_Ser28 (4HPO) (7). V_H_ is green; V2 is magenta. **(D)** In affinity-matured CH58, the mutation V_H_Ser28→Arg results in formation of a bidentate salt bridge with V2 Asp180. V_H_ is teal. This residue was not visible in **(C)** due to disorder in the C-terminus of the V2 peptide.

Antibody CH58 was isolated from a participant in the RV144 HIV vaccine efficacy trial. Like most bNAbs elicited in response to HIV-1 infection, CH58 is highly mutated (7). The structure of affinity-matured CH58 in complex with an Env V2 peptide showed that this bNAb targets V2 residue Lys169, which is a site of vaccine-induced immune pressure. Structures have also been determined for the putative germ line precursor of CH58 in unliganded form and bound to the V2 peptide (8). A comparison of these structures revealed that affinity maturation of CH58 is driven by the formation of two new salt bridges linking V_L_CDR1Asn31→Asp and V_H_CDR1Ser28→Arg to Lys171 and Asp180 of V2, respectively (Figure 1) (8). In addition, V_L_CDR3 in the unbound germ line precursor adopts a different conformation in the CH58–V2 complex, implying flexibility. By contrast, the conformation of V_L_CDR3 in the CH58–V2 complex is nearly identical to that in unbound matured CH58. Such preorganization of the CH58 paratope into a configuration more suitable for binding V2, accompanied by rigidification of V_L_CDR3 to lower the entropic cost of complex formation, further contributes to the 2,000-fold affinity increase during maturation. Paratope preorganization and rigidification have also been described for the CH65 lineage of anti-influenza virus HA antibodies (21), underscoring the general utility of these mechanisms for improving affinity (26–29).

A related study used hydrogen/deuterium exchange in combination with mass spectrometry (HDX/MS) to investigate how affinity maturation alters the dynamics of bNAbs against HIV-1 Env (12). Importantly, the high variability and constantly evolving nature of HIV-1 Env distinguish this conformationally dynamic glycoprotein from the static model antigens used in studies of affinity maturation prior to NGS (23–26, 30). HDX/MS directly measures local protein dynamics by monitoring backbone amide deuterium uptake when the protein is diluted into a solution of D_2_O. Dynamic regions of a protein take up deuterium more rapidly than stable regions. HDX/MS was used to compare the local dynamics of the predicted germ line and affinity-matured forms of two bNAbs (VRC03 and VRC-PG04) specific for the CD4 binding site of HIV-1 Env (12). In both cases, the paratopes of the matured bNAbs were less dynamic overall that those of their germ line counterparts, in agreement with previous evidence that paratopes become more rigid during the maturation process (28, 30, 48). Surprisingly, however, the largest decreases in dynamics occurred at the periphery of the paratopes, at sites adjacent to Env glycans, rather than at primary Env-contacting sites. A similar pattern was observed for a bNAb (CAP256-VRC26) specific for the V1/V2 quaternary epitope of HIV-1 Env. This stabilization of the paratope periphery may serve to minimize potential clashes with nearby Env glycans, while maintaining critical binding interactions mediated by the center of the paratope. It is probably not coincidental that a similar focus of affinity maturation on sites peripheral rather than central to the interface with antigen was also observed for anti-HEL antibodies (30).

**Figure 2:**
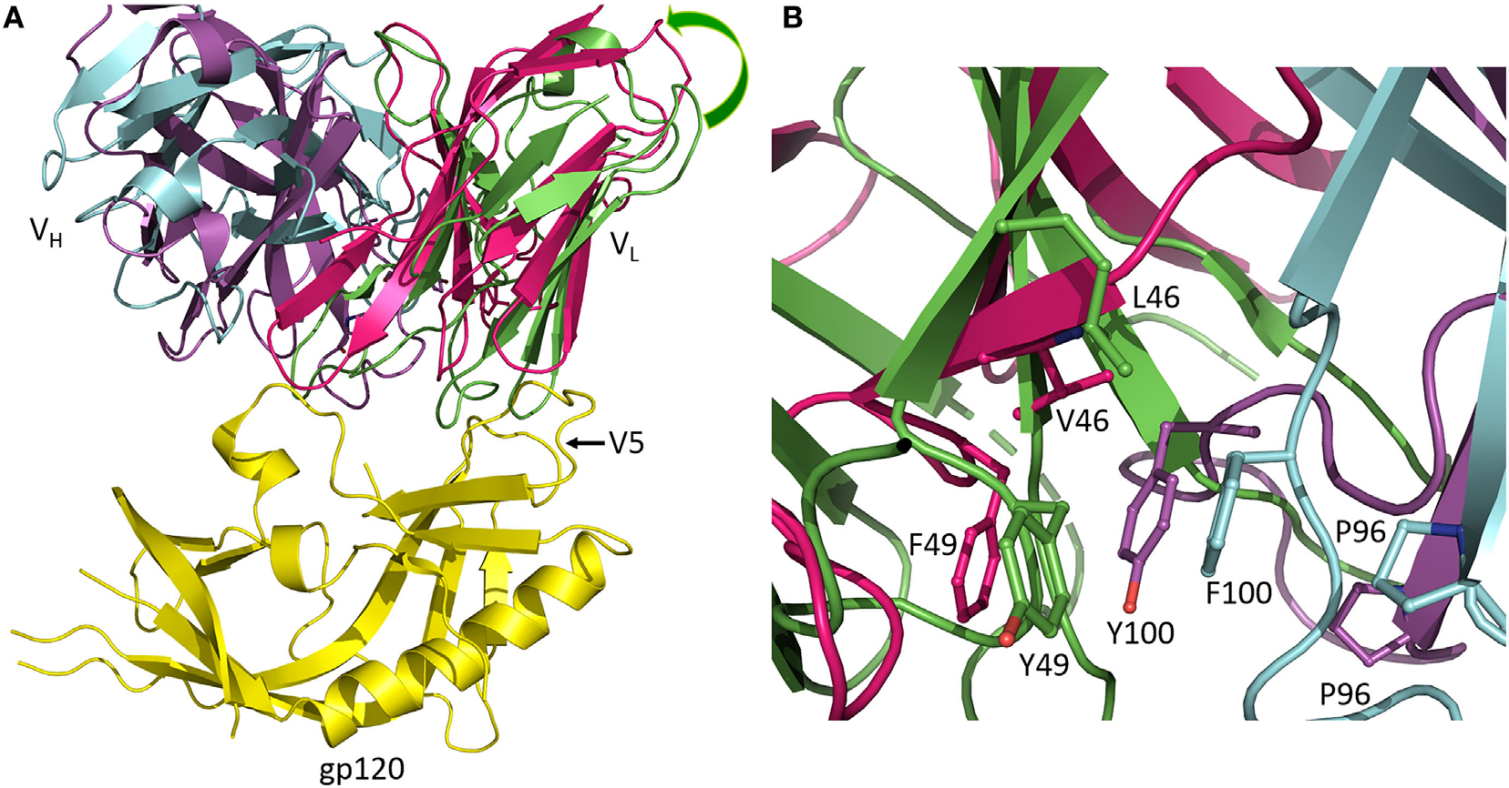
Reorientation of V_L_ and V_H_ domains in response to viral escape mutations. **(A)** Superposition of the germ line precursor of CH103 (V_L_ and V_H_ domains are green and cyan, respectively) (4QHL) (14) onto matured CH103 (V_L_ and V_H_ domains are red and magenta, respectively) in complex with envelope glycoprotein gp120 (yellow) (4JAN) (13). During affinity maturation, a shift occurred in the orientation of V_L_ with respect to V_H_. The shift is an adaptation to insertions in the gp120 V5 loop during infection. Movement of V_L_ away from gp120 enables accommodation of the V5 insertion. **(B)** Close-up view of the V_L_/V_H_ interface in the vicinity of the V_L_Leu46→Val mutation showing changes in interdomain contacts and rearrangement of V_H_CDR3.

Antibodies of the CH103 lineage block the CD4 binding site of HIV-1 *via* interactions dominated by V_H_CDR3 (13). Affinity maturation of CH103 is associated with mutations in both contacting and non-contacting residues, including framework (FR) residues distant from the interface with Env. Structural analysis of the putative germ line precursor of CH103 and of two intermediates in the maturation pathway revealed a shift in the relative orientation of the V_L_ and V_H_ domains during evolution of the CH103 lineage, corresponding to a root mean squared deviation in α-carbon positions of 2.1 Å (Figure 2A) (14). This shift is mediated by several mutations at the V_L_/V_H_ interface, including a leucine-to-valine substitution at FR position V_L_46 that not only contributes to reorienting the V_L_ domain but also to reconfiguring V_H_CDR3 (Figure 2B). Although not as important as the V_L_Leu46→Val mutation, the neighboring V_L_Tyr49→Phe and V_H_Phe100→Tyr mutations, which are also located in the V_L_/V_H_ interface, may further contribute to V_L_/V_H_ reorientation. Most likely, V_L_/V_H_ reorientation occurred in response to insertions in the V5 loop of Env during infection, which had allowed the virus to escape neutralization by progenitors of CH103. Displacement of V_L_ away from V5 allowed accommodation of insertions in V5 without steric hindrance (14). In addition, the conformation of V_H_CDR3 in the germ line precursor of CH103 is incompatible with gp120 binding, at least as observed in the CH103–gp120 complex (13), thereby necessitating rearrangement of this loop during the maturation process (14).

In sharp contrast to bNAbs against HIV-1, which are highly mutated, the potent human anti-Middle East respiratory syndrome coronavirus antibody m336 is almost germ line, with only one somatic mutation in the H chain (49). The structure of m336 in complex with the MERS-CoV receptor-binding domain showed that the IGV1-69-derived H chain contributes >85% of the binding surface. The subnanomolar affinity of m336 despite the near absence of somatic mutations results from direct interactions with germ line-encoded V_H_CDR2 and V_H_ framework region 3 (V_H_FR3) residues and with recombination-generated residues in the V_H_DJ_H_ junction (49). Like m336, the anti-influenza HA bNAb CR6261 uses the IGV1-69 V_H_ gene segment (47). Virus neutralization depended solely on the H chain, and only seven somatic mutations in V_H_CDR1 and V_H_FR3 were required for maximum affinity.

To systematically dissect the contribution of mutations in FR residues to affinity maturation, deep mutational scanning was applied to the anti-vascular endothelial cell growth factor antibody G6.31 (50). A number of FR mutations at positions distal to the CDRs were found to improve the affinity and/or thermostability of G6.31. In particular, the FR mutation V_L_Phe83→Ala, which is ~25 Å away from the antigen-binding site, increased both affinity and stability by altering the orientation of the constant domains (C_L_ and C_H_1) relative to V_L_ and V_H_, as well as the orientation of V_L_ to V_H_ (50). As measured by HDX/MS, the V_L_Phe83→Ala mutation modulated the interdomain conformational dynamics of antibody G6.31. Furthermore, analysis of > 5,000 human V_L_ sequences showed that somatic mutations occur frequently at position 83, strongly suggesting its biological relevance in repertoire selection.

### Affinity Maturation of Glycan-Binding Antibodies

Besides high sequence variability, another feature of HIV-1 Env that distinguishes it from model antigens such as HEL used in previous studies of affinity maturation is glycosylation. Indeed, extensive N-glycosylation masks much of the Env protein surface from antibody recognition. Nevertheless, a number of potent bNAbs have been discovered that penetrate this glycan shield and engage both carbohydrate and protein antigenic determinants (51, 52). In the most thoroughly studied example to date, the PGT121 family of bNAbs was shown to bind to N-glycans located in a high-mannose patch centered on the highly conserved Asn332 glycan and to protein elements at the base of the V3 loop (15, 16).

**Figure 3:**
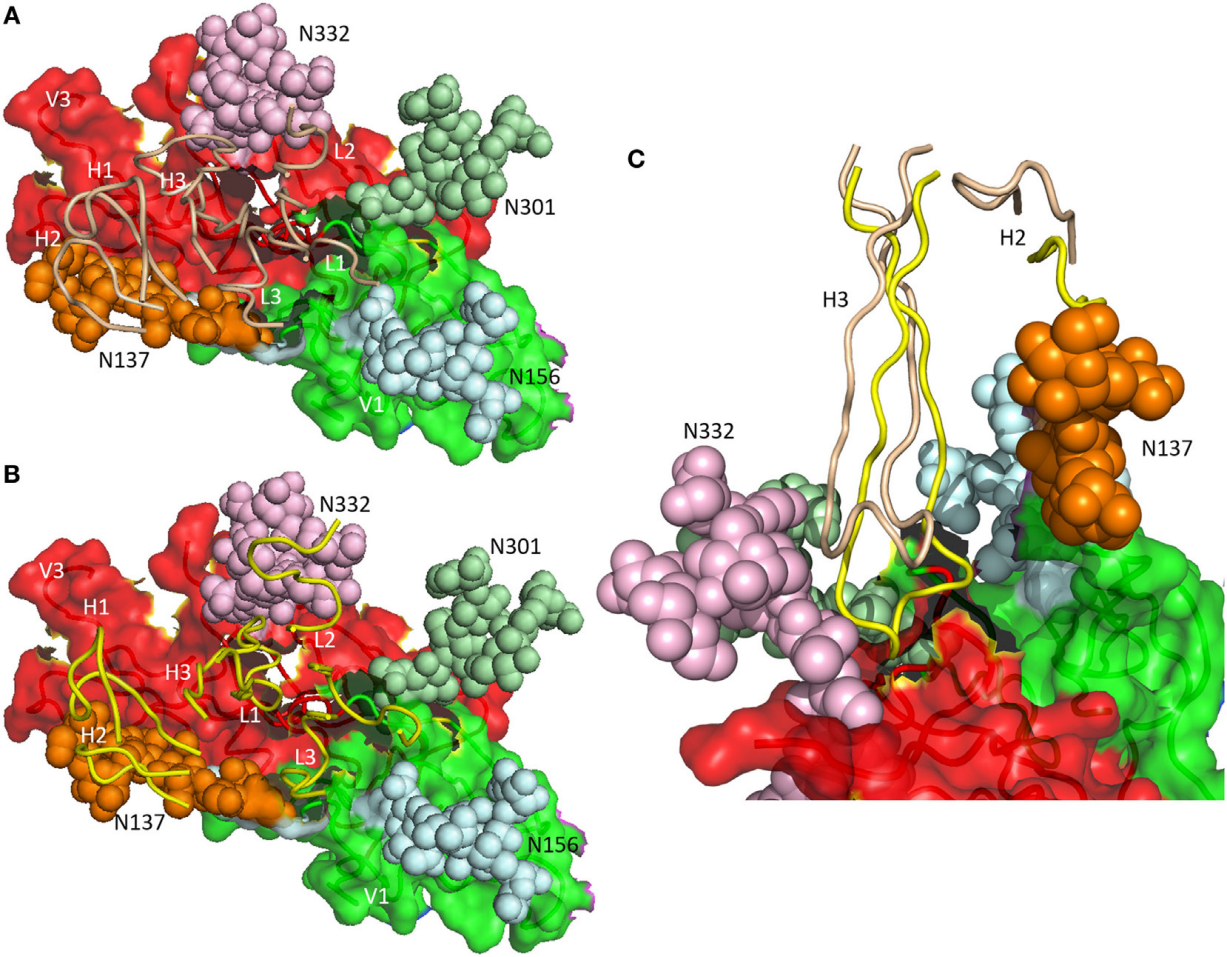
Differential glycan recognition by affinity-matured antibodies. **(A)** Positions of complementarity-determining region (CDR) loops (wheat) of matured antibody PGT124 on envelope glycoprotein (Env) gp120 (4R2G) (16). V_L_ CDR loops are labeled L1–L3; V_H_ CDR loops are labeled H1–H3. The V1 (green) and V3 (red) regions of gp120 are depicted as molecular surfaces. Glycans are drawn as spheres and labeled N137, N156, N301, and N332. The PGT124 epitope is composed of V1, V3, and the N332 glycan. **(B)** Positions of CDR loops (yellow) of matured antibody PGT122 on Env gp120 (4NCO) (15). The PGT122 epitope is composed of V1 and V3, as well as all four glycans at N137, N156, N301, and N332. **(C)** V_H_CDR3 of PGT122 (yellow) contacts both the N137 and N332 glycans. V_H_CDR3 of PGT124 (wheat) contacts only the N332 glycan due to a shift in position relative to V_H_CDR3 of PGT122.

As revealed by NGS and X-ray crystallography, the putative germ line precursor of the PGT121 family splits into two evolutionary branches that differ considerably in how they interact with Env glycans (Figures 3A,B). One branch, exemplified by PGT124, contacts only the Asn332 glycan (16), whereas the other branch, exemplified by PGT122, also contacts the Asn137, Asn156, and Asn301 glycans (15). PGT124 and PGT122 employ a common set of CDR residues to contact the Asn332 glycan and V3 loop, nearly all of which are also found in the germ line precursor. This conservation suggests that a critical event in triggering the antibody response is simultaneous recognition of both carbohydrate and protein determinants.

Antibodies PGT124 and PGT122 share similar binding site architectures with long V_H_CDR3 loops that pack against the entire length of the Asn332 glycan, thereby penetrating the glycan shield to reach the Env protein surface below (Figure 3C) (16). In both antibodies, as well as in the germ line precursor, a closed face on one side of the paratope engages the Asn332 glycan. A second, open face differs between PGT124 and PGT122. For PGT124, the open face enables avoidance of neighboring glycans through a shift of V_H_CDR3 away from its equivalent position in PGT122 (Figure 3C). For PGT122, by contrast, mutations during affinity maturation result in productive interactions between V_H_CDR2 and the Asn137 glycan. In agreement with the structural data, deletion of the Asn332 glycan abolished PGT124 neutralization of nearly all HIV-1 isolates, whereas deletion of neighboring glycans had little or no effect (16). Conversely, PGT122 is less reliant on the Asn332 glycan than PGT124 for virus neutralization because of its ability to utilize alternative glycans at Asn137 and Asn301 to achieve high-affinity binding.

Recently, NGS of HIV-1 longitudinal cohorts has been performed to investigate coevolution of HIV-1 Env and antibodies targeting this glycoprotein and to identify viral variants that initiate maturation of bNAbs (18, 53, 54). These studies have shown that viral escape generates a pool of diverse epitope variants and that somatic hypermutation, acting in parallel, creates antibodies with differential ability to neutralize these variants. Thus, antibodies from the CAP256 and PCT64 lineages, which were isolated independently from two different HIV-1 infected African patients, both target the V2 apex epitope of the Env trimer (18, 53, 54). The crystal structure of bNAb PCT64-35B, isolated at 35 months postinfection, showed that the somatically mutated 25-residue V_H_CDR3 loop adopts a β-hairpin conformation that projects above the other CDRs (18). The extended conformation and anionic character of this V_H_CDR3, which contains at least one sulfotyrosine, probably enable this loop to penetrate between the glycans that shield the V2 apex epitope to contact the positively charged V1/V2 protein surface of the Env trimer. Moreover, in the PCT64 donor, Env glycoform heterogeneity may have played a role in activating B cell precursors of the PCT64 lineage by allowing germ line antibody binding to early Env trimer forms lacking complex or hybrid glycans at key positions (18).

According to the polyreactivity hypothesis, B cells initially produce germ line antibodies with conformationally flexible combining sites that are able to recognize diverse antigens with low affinity (12, 28, 48). In this way, the immune system can respond to an enormous variety of potential antigens, whose numbers dwarf theoretical estimates of the clonal diversity of germ line antibodies. As described earlier, studies of immune responses to haptens, model proteins, and HIV-1 Env have shown that affinity maturation generates antibodies with higher affinity and specificity than germ line antibodies, at least in part through rigidification of the paratope. However, a recent study of glycan-specific antibodies has suggested an alternative pathway for antibody evolution that is distinct from the polyreactive germ line pathway (55).

A combination of glycan microarrays and molecular dynamics (MD) simulations was used to investigate the affinity maturation of two antibodies (3F8 and ch14.18) specific for the ganglioside GD2, a tumor-associated carbohydrate antigen overexpressed in various cancers, notably neuroblastoma and melanoma (55). Surprisingly, the putative germ line antibodies, although of lower affinity than their matured counterparts, were just as highly selective for CD2 as the matured antibodies when screened against hundreds of glycans and thousands of proteins. Possible reasons for this lack of observable polyreactivity were investigated by MD simulations of germ line and affinity-matured 3F8 and ch14.18 (55). These simulations revealed that, rather than becoming more rigid, the binding sites of the matured anti-G2 antibodies showed an increase in flexibility relative to the binding sites of the germ line antibodies. Most likely, affinity maturation of 3F8 and ch14.18 was enthalpically driven by an increase in direct or water-mediated hydrogen bonds to G2, rather than entropically driven by a decrease in binding site flexibility. The high selectivity of germ line antibodies to the ganglioside G2, compared to the polyreactivity of antibodies to other antigens (12, 28, 48), may reduce the risk of developing autoimmune diseases such as Guillain–Barré syndrome, which is associated with antibodies to the structurally related ganglioside GM2 (56).

### Indels in Antibody Affinity Maturation

Multibase in-frame indels are introduced during somatic hypermutation in germinal center B cells along with point mutations (57). As revealed by NGS, the frequency of indels among total somatic mutations in normal human B cell repertoires is low (~2%) (58). In sharp contrast, ~40% of bNAbs against HIV-1 Env contain indels, ranging in size from 1 to 11 amino acids (59). Indels are the primary means by which antibodies can effect large changes in steric volume comparable to those associated with addition or removal of glycans. Indeed, the role of indels in bNAb maturation is to accommodate glycans, penetrate the glycan shield, and/or increase the number of interfacial contacts.

A distinguishing feature of VRC01-class bNAbs that target the CD4 binding site of Env is a deletion of two to six amino acids in V_L_CDR1 (9, 10). The function of this deletion is to avoid a steric clash with the Asn276 glycan of loop D of gp120, such that reversion of the deletion to germ line markedly diminished binding. Notably, the V_L_CDR1 deletion appears across multiple lineages of VRC01-class bNAbs from different individuals, suggesting that there are few other viable solutions to fitting an antibody into the CD4 binding site of Env.

**Figure 4:**
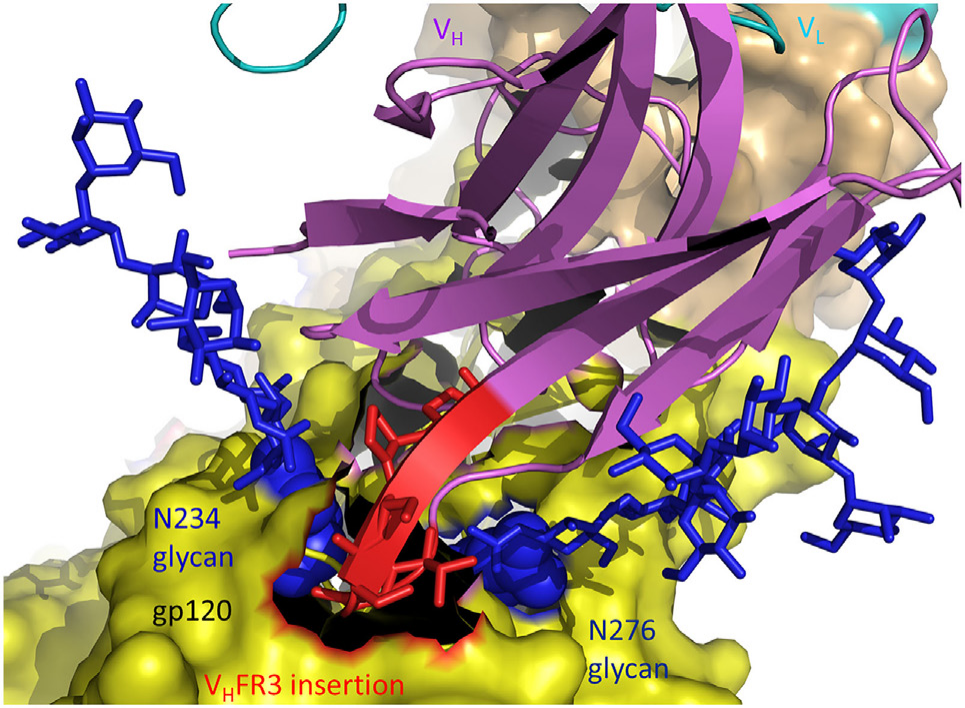
Role of insertions in antibody affinity maturation. An insertion of five amino acids in V_H_ framework region 3 (V_H_FR3) of matured antibody 8ANC195 enables V_H_FR3 to extend between the N234 and N276 glycans of envelope glycoprotein (Env) gp120 and contact protein beneath the glycan shield (5CJX) (20). V_H_ and V_L_ are magenta and cyan, respectively. The insertion in V_H_FR3 is red. Env gp120 (yellow) is drawn as a molecular surface. Glycans are represented as blue sticks.

The structure of antibody 8ANC195 in complex with trimeric Env showed that this bNAb recognizes an epitope that spans the gp41 and gp120 Env subunits (20). An insertion of five amino acids in V_H_FR3 extends between the Asn234 and Asn276 glycans of gp120, establishing productive interactions with both glycans and enabling 8ANC195 to penetrate the glycan shield to contact the protein surface of Env (Figure 4). Like 8ANC195, bNAb 35O22 binds trimeric Env at the gp41–gp120 interface (60). An insertion of eight amino acids in V_H_FR3 projects into the cleft between the Env subunits, resulting in additional stabilizing contacts with antigen that increase the neutralization potency of the matured antibody compared to its germ line precursor.

## Conclusion

Structural studies of antibody affinity maturation spanning nearly 30 years have identified a diversity of biophysical mechanisms underlying this prototypical example of molecular evolution. These include improved shape complementarity at the interface with antigen, increased buried surface area upon complex formation, additional interfacial polar or hydrophobic interactions, preorganization or rigidification of the antigen-binding site, and V_L_/V_H_ reorientation. Over the last 5 years, NGS has allowed the reconstruction of antibody clonal lineages in immune responses to viral pathogens, mainly HIV-1, which was not possible previously. These remarkable studies have revealed how antibodies evolve to penetrate the glycan shield of HIV-1 Env and how the virus in turn evolves to escape neutralization. New insights into the coevolution of viruses and antiviral antibodies will come from NGS of both Env variants and antibodies targeting these variants in well-documented longitudinal cohorts of African HIV-1 patients, as demonstrated recently (18, 53, 54). Although HIV-1 has so far been the primary focus of studies using NGS and single-cell analysis to identify germ line progenitors and intermediates along antibody maturation pathways, the future application of these methods to other pathogens, such as dengue virus and Ebola virus, will undoubtedly uncover new structural strategies for generating high-affinity binders to bolster host immune defense.

It is becoming increasingly evident that the contribution of somatic hypermutation to bNAb development must be considered in designing vaccine immunogens for different viral pathogens. At one extreme, bNAbs against some viruses, such as MERS-CoV (49) and hepatitis C virus (61), appear to exist naturally with relatively few somatic mutations. Importantly, somatic mutations were found not to be required for binding of germ line ancestors of these bNAbs to their viral targets, which may facilitate elicitation of effective vaccine responses. At the other extreme, bNAbs against viruses such as HIV-1 are highly somatically mutated and generally require years to develop in infected individuals. Furthermore, germ line precursors of these bNAbs often exhibit little or no detectable affinity for HIV-1 Env, making elicitation of such antibodies by vaccination a formidable challenge (62). Current strategies to overcome this roadblock involve initial activation of naïve mature B cells expressing germ line B cell receptors with engineered germ line-binding immunogens, followed by sequential vaccinations with immunogens designed to bind intermediate antibodies in order to guide the immune system through complex maturation pathways that ultimately lead to antibodies with specific somatic mutations conferring high affinity and neutralization potency (63–66).

## Author Contributions

AM and RM conceived and wrote the manuscript.

## Conflict of Interest Statement

The authors declare that the research was conducted in the absence of any commercial or financial relationships that could be construed as a potential conflict of interest.
